# Opportunities for synthetic data in nature and climate finance

**DOI:** 10.3389/frai.2023.1168749

**Published:** 2024-01-09

**Authors:** Nataliya Tkachenko

**Affiliations:** ^1^Smith School of Enterprise and the Environment, University of Oxford, Oxford, United Kingdom; ^2^UK Centre for Greening Finance and Investment, University of Oxford, Oxford, United Kingdom; ^3^The Alan Turing Institute, Finance and Economics, The British Library, London, United Kingdom

**Keywords:** synthetic data, spatial finance, adaptation analytics, ESG, generative AI, AI ethics, nature finance, climate finance

## Abstract

This paper delves into the intricacies of synthetic data, emphasizing its growing significance in the realm of finance and more notably, sustainable finance. Synthetic data, artificially generated to simulate real-world data, is being recognized for its potential to address risk management, regulatory compliance, and the innovation of financial products. Especially in sustainable finance, synthetic data offers insights into modeling environmental uncertainties, assessing volatile social and governance scenarios, enhancing data availability, and protecting data confidentiality. This critical review attempts first ever classification of synthetic data production methods, when applied to sustainable finance data gaps, elucidates the methodologies behind its creation, and examines its assurance and controls. Further, it identifies the unique data needs of green finance going forward and breaks down potential risks tied to synthetic data utilization, including challenges from generative AI, input quality, and critical ethical considerations like bias and discrimination.

## 1 Introduction

Synthetic data generation is increasingly regarded as a paradigm shift in quantitative finance and refers to data that is artificially generated rather than being derived from real-world events (Drechsler and Reiter, 2012; Stodden, 2015; Burgard et al., 2017). Despite the common assumption, this data is not represented by random numbers; Rather, it is usually carefully constructed to simulate real-world data in terms of structure, statistics, and relevance. Various modern algorithms, including those rooted in deep learning and other advanced computational methods, have made the generation of high-quality synthetic data feasible and increasingly accurate. Whilst use of synthetic samples and approximated simulations have been widely used in various fields since early 90s, especially theoretical physics, clinical medicine, geology, astrophysics, organic chemistry and other fields, real applications of synthetic data in finance did not really emerge until late 2000s, which coincided with the growing interest in machine learning algorithms within trading and portfolio optimisation realms.

The growing importance of simulated data in finance was factored by several conditions. Primarily, the trend was driven by requirements for more resilient risk management frameworks (White, 2021; Heim, 2022). Thus, traditional financial models are known for their over-reliance on historical data to predict future trends; However, unprecedented events like the 2008 financial crisis or the COVID-19 pandemic underscored some serious limitations of such dependencies. Synthetic data therefore emerged as a suitable alternative for modeling extreme but plausible scenarios to test the resilience of financial systems and instruments. Secondly, increasing requirements of regulatory compliance put pressure on financial institutions to stress-test their portfolios under various scenarios; Realistic synthetic datasets enabled modeling diverse scenarios without compromising the confidentiality of real customer data. And finally, last but not least role was played by accelerated innovation in financial products. With the rise of various FinTechs, an urgent need emerged to iterate and experiment rapidly, and synthetic data allowed firms to test new algorithms and financial products without waiting for real-world data to be produced and accumulated (Fienberg, 1994; Burgard et al., 2017).

### 1.1 Growing significance of synthetic data in sustainable finance

Sustainable finance in its broadest terms refers to the inclusion of environmental, social, and governance (ESG) considerations in investment decisions, aiming to achieve long-term returns while also addressing societal challenges (Migliorelli, 2021; Papenbrock et al., 2021). Whilst data scarcity is arguably the most recognized problem in green finance research, predominantly due to a lack of tradition of externalities' accounting, synthetic data is hardly mentioned in this context at all. Moreover, since data scarcity is often associated with lack of expertise in data formatting, the main effort to tackle it was directed toward collecting high resolution real data entries, rather than exploring the potential and usability of synthetic variables and their proxies.

Nevertheless, increasing demand from regulators and civil societies for higher quality of models of environmental uncertainties highlighted another aspect of the ‘scarce data problem' in sustainable finance - notably, absolute lack of it (Dye et al., 2021; Irvine-Broque and Dempsey, 2023). Thus, growing climate change concerns revealed that there is an absence of historical data for some regions or time periods that can capture potential future realities and provide reliable representative projections into the future. And at the same time, wealth of methodological traditions in synthetic data, accumulated for and by other disciplines, suddenly opened up opportunities not only for modeling potential climate crises (Koh et al., 2020; Van Horn et al., 2021), but also turned out to become quite instrumental in helping sustainable finance professionals understand potential environmental risks (and opportunities) in their portfolios.

Timely and accurate assessment of social and governance scenarios appeared as another challenge for sustainable financial institutions. Just as with environmental challenges, the social and governance landscapes are extremely dynamic, and synthetic data, derived from various unstructured social web data sources, became a quick an easy solution for simulations of various possible futures, such as political upheavals, labor strikes, or governance failures, providing insights into how they might impact financial returns (Keen, 2021; Barnes, 2022).

Data availability provisioning and enhancement for many ESG factors, especially in emerging markets, where there is a dearth of comprehensive and reliable data, synthetic data opened up opportunities to fill these gaps, allowing for a more holistic assessment of sustainable investment opportunities. This also allowed to address additional constraints, specifically growing data ethics standards. Protection of confidentiality and privacy is a well known ‘old data tradition', and as ESG investing often considers sensitive information (including company's internal governance practices or activities near environmentally sensitive areas), synthetic data can become instrumental in information sharing protocols without revealing proprietary or confidential details (LaBella et al., 2019; Triantafyllou et al., 2020).

It has been recognized that now is the high time for new models of collaboration between science and finance to enhance climate and nature scenarios (Dietz et al., 2021; Kahn et al., 2021; Warren et al., 2021). Much attention is drawn to the challenges of the current generation of climate scenarios used by banks (Kemp et al., 2022), insurers and pension funds to manage climate risks, specifically on how these scenarios underestimate risks (Zscheischler et al., 2018; Ranger et al., 2021; Pitman et al., 2022). Whilst recent research shows that many financial institutions recognize the issues and are working to rectify this (the two-thirds agreeing there are material sources of risk not captured in current scenarios), the role of missing/inadequate data is poorly acknowledged and there is a little appreciation in the scientific community of how synthetic data could address many of those issues. In the scope of this paper we make an argument that not only it can help facilitating better risk assessment and product innovation but also can ensure that the finance industry navigates future uncertainties and complexities with a lot more confidence.

### 1.2 How regulators support use of synthetic data in finance and sustainable investing

Many regulatory bodies around the world have shown interest in FinTech and data innovations, however, explicit endorsements or guidelines on synthetic data are still developing. Regular consultations with these bodies or checking their latest publications provide the most up-to-date stance on the topic.

- Thus, in the US, Securities and Exchange Commission (SEC)^1^ for securities and Commodity Futures Trading Commission (CFTC)^2^ have not yet explicitly endorsed the widespread promotion of synthetic data, they nevertheless pay a very close attention to this proliferating method since the U.S. has numerous FinTech and tech firms exploring the potential of synthetic data in finance.
- Canadian regulatory bodies (FCAC)^3^ are increasingly interested in FinTech innovations, though widespread use or endorsement of synthetic data is still in nascent stages.
- Australian Securities and Investments Commission (ASIC)^4^ is known for its progressive stance on FinTech, and they have shown interest in various technologies, including the potential use of synthetic data.
- European Securities and Markets Authority (ESMA)^5^ currently focuses on the development of a common rulebook for European Union (EU) financial markets. And whilst synthetic data is not a prominent agenda yet, various EU member states have individual FinTech initiatives that might delve into it in the near future.
- Monetary Authority of Singapore (MAS)^6^ has been at the forefront of FinTech innovation for a few years now, and they have already explored various data solutions in this space, including the potential of synthetic data for the financial industry.
- Securities and Exchange Board of India (SEBI)^7^ has shown increasing interest in FinTech innovations, though the widespread discussion of synthetic data is still emerging.
- China Securities Regulatory Commission (CSRC),^8^ with the rapidly growing FinTech landscape in the country, is exploring various data-driven solutions, however, the explicit stance of CSRC on synthetic data is not widely documented yet.
- Financial Services Agency (FSA) of Japan^9^ has been proactive in embracing FinTech innovations; And although not explicitly focused on synthetic data, the FSA is demonstrably keen on technologies enhancing financial services.
- In South Africa, Financial Sector Conduct Authority (FSCA)^10^ is paying significant attention to the customer protection and ensuring a stable financial market technologies, and are expected to look into synthetic data as FinTech grows in the region.
- In Brazil Comissão de Valores Mobiliários (CVM),^11^ who are primarily concerned with securities market regulations, are also increasingly engaging with FinTech, with the growing prospects of potentially including synthetic data discussions in the future.
- In the UK, the Financial Conduct Authority (FCA) introduced the first ever Expert Group on Synthetic Data, which functions as part of the broader Innovation Advisory (IAG).^12^ It has both fixed and rotating members, and it is covering a broad set of topics, including the use of synthetic data in financial services, alternative approaches to future TechSprints and future-proofing of innovation services. The IAG primarily supports the FCA's innovation work, and the group can discuss wider topics which contribute to the FCA's strategic commitment to promote competition and positive change. Under their guidance, the priority cases have been identified as ethical-by-principle financial use cases (heavily reliant on tokenisation/pseudonymisation), which should be further extended within Permanent Sandbox environment.^13^ Synthetic data generation methods should be fully documented to maintain transparency and reproducibility, and FCA currently considers how use of synthetic data can help to meet and comply with AI ethics principles and requirements, specifically model fairness, preparation of the data needs to meet representativeness and lack of bias, and GDPR compliance via tokenisation (privacy and security). Since the exact use cases have not been published yet, it is therefore difficult to estimate to what extent synthetic data work will extend toward regulatory requirements of sustainable reporting frameworks, and how these two agendas will be co-evolving within FCA's future scopes of activities.

## 2 Detailed data requirements for green finance (and how synthetic data can help to meet them)

### 2.1 Synthetic data definitions and applications

Despite its relatively low profile, which arguably became more prominent recently, alongside the proliferation of generative AI models and their ever-growing input data requirements, synthetic data has always been a crucial topic in contemporary financial analytics and research. Conceptually, synthetic data deviates from real data as its byproduct, inheriting its major statistical properties. This imitation of real data enables synthetic data to serve as an efficient proxy, the efficacy of which is determined by its utility.

One of the foundational strengths of synthetic data lies in its ability to bridge gaps in the financial sector where real data accessibility is either hindered by confidentiality or economic constraints. Acquiring vast quantities of historical market data within the financial industry often comes with substantial costs. Furthermore, leveraging customer financial transactions is fraught with challenges, primarily due to the sensitive nature of personal financial information. This sensitivity amplifies when data sharing extends beyond organizational boundaries to include external analysts.

To alleviate these challenges, synthetic data emerged as a pivotal solution in two significant ways: by provisioning efficient data access and by enhancing analytical competence. By circumventing the need for real data, especially when the latter is sensitive or confidential, synthetic proxies can not only safeguards personal financial information but also ensure that data-driven analytics aren't hampered by accessibility issues. When increased competence required, synthetic data can also facilitate the creation of standardized data benchmarks, a tool that proves invaluable when assessing the quality and reliability of data or models procured from third-party vendors.

In sustainable finance, where analysts often grapple with scenarios where real data is either non-existent or not standardized for specific financial applications, synthetic data can address this issue by enabling analysts to simulate data in scenarios where real-world data collection is either exorbitantly costly or logistically unfeasible. Beyond its cost and logistical advantages, synthetic data also shines in its capacity to represent edge or rare cases, scenarios where real-world data collection may border on the unethical or is simply too challenging. Another prevalent issue in data analytics is the presence of unlabeled real data; Thus, manually labeling such data is not only tedious but also susceptible to errors, hence synthetic data can bypass this issue by offering pre-labelled datasets for downstream applications. Given the increasingly diverse and complex analytical landscapes, such as modeling the macro-economic implications of various climate change scenarios or nature risks and dependencies, synthetic data has a string potential to aid analysts in validating their models and assumptions. This validation is crucial, ensuring that derived results closely mirror potential real-world outcomes.

Broadly classifying, synthetic data can be categorized into three types: [1] Derived from real datasets (this type capitalizes on actual data, deriving its statistical properties to generate a synthetic counterpart); [2] Independent of real data (this variant is generated without leveraging any real datasets, often used when real data is either unavailable or irrelevant); [3] Hybrid (a fusion of the above two types, this category often seeks to combine the strengths of both, offering a more holistic dataset). Each type finds its niche application across diverse financial use cases, and the choice among them hinges on the specific requirements of the task at hand, and the method of data synthesis best suited to achieve optimal results (which will be discussed in the following chapters of this paper).

### 2.2 Major regulatory frameworks driving data requirements in sustainable finance

**Figure 1:**
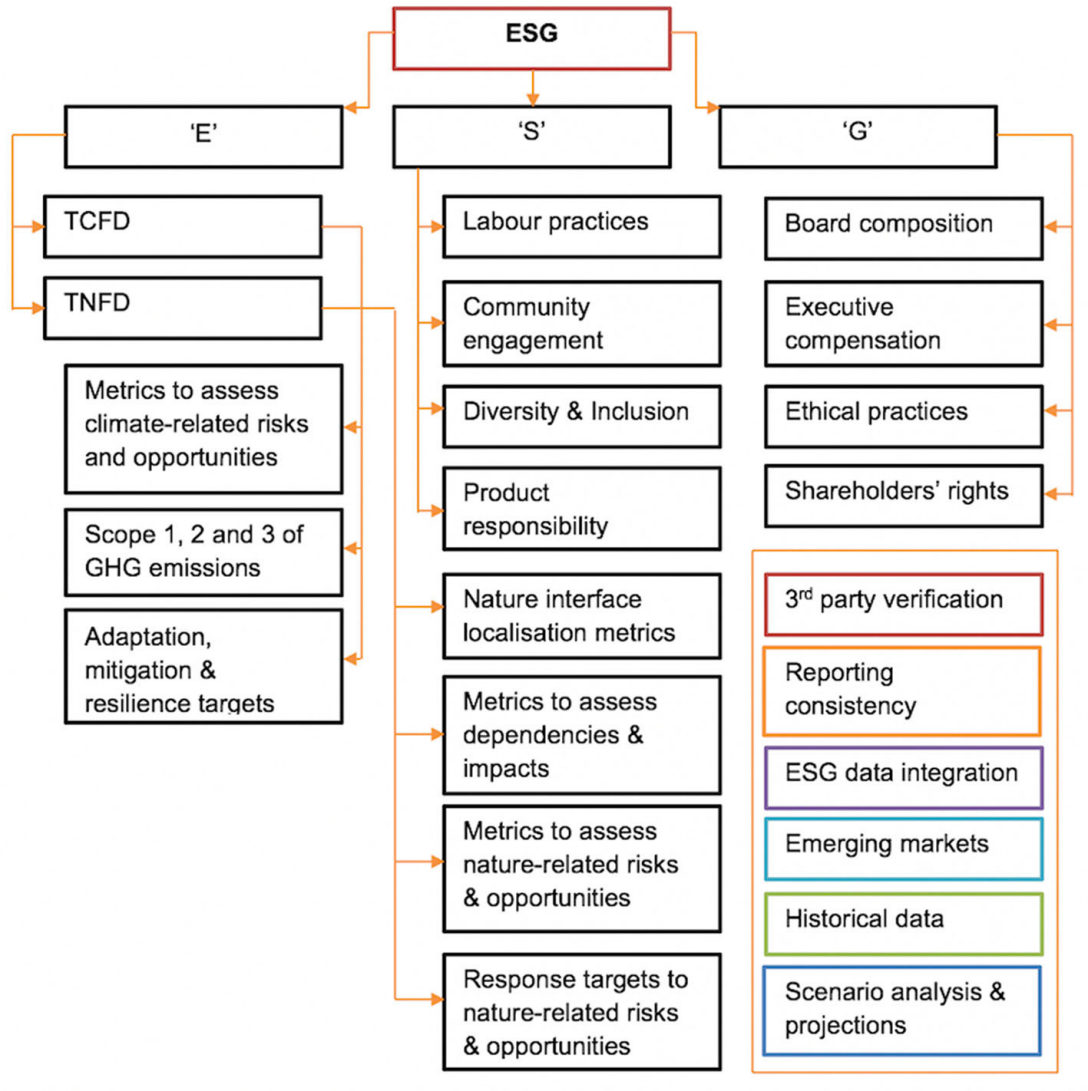
Emerging information and metrics needs in the field of sustainable finance, driven by both regulatory and innovation requirements.

Whilst significant body of research literature exists Migliorelli (2021); Papenbrock et al. (2021), covering regulatory, statutory and supervisory green transition frameworks, their origins and inter-dependencies, there is currently very little information about metrics requirements described in simple and accessible data flows format. Five major international reporting frameworks are SASB, GRI, UN SDGs, TCFD^14^ and emerging TNFD, and since first three have been extensively covered in research literature across their data requirements, case studies and ethical implications, hence we will focus here predominantly on the information deficiencies for TCFD/TNFD frameworks (Amel-Zadeh and Serafeim, 2018; Grewal et al., 2019; Kotsantonis and Serafeim, 2019; Porter et al., 2019; Karageorgiou and Serafeim, 2021; Christensen et al., 2022; Pollard and Bebbington, 2022; Serafeim and Yoon, 2022a,b). And in attempt to make sense of their data typologies, we propose the following structure below (Figure 1).

**[1]** ESG is often mentioned interchangeably along sustainable finance; The term itself stands for Environmental, Social, and Governance indicators and it is a broad framework used by investors and other stakeholders to assess a company's performance and risk exposure in these three areas. ESG is becoming increasingly important in the financial world, as there's a growing recognition that ESG factors can have a significant impact on a company's long-term value. **[2]** TCFD (Task Force on Climate-related Financial Disclosures) and **[3]** TNFD (Task Force on Nature-related Financial Disclosures) are both initiatives aimed at providing guidance and standards for companies to report on specific environmental risks (Dye et al., 2021; HOEKSTRA, 2022; Rudman et al., 2022; Chiu et al., 2023; Irvine-Broque and Dempsey, 2023; Lee et al., 2023; Adams et al., 2024).

The Task Force on Climate-related Financial Disclosures (TCFD) was established by the Financial Stability Board (FSB) to develop consistent climate-related financial risk disclosures for use by companies, banks, and investors. The recommendations are organized around four thematic areas: **(i)** Governance (company's governance structures around climate-related risks and opportunities); **(ii)** Strategy (actual and potential impacts of climate-related risks and opportunities on the organization's businesses, strategy, and financial planning); **(iii)** Risk Management (processes used by the organization to identify, assess, and manage climate-related risks); **(iv)** Metrics & Targets (metrics and targets used by the organization to assess and manage relevant climate-related risks and opportunities).

The TCFD lists three recommended disclosures under Metrics & Targets, of which the second is focused on the disclosure of greenhouse gas emissions (Scope 1, Scope 2, and, if appropriate, Scope 3 greenhouse gas (GHG) emissions), and guidance on physical risk disclosures focuses on the first and third disclosures. Metrics & Targets are at the core of a climate-related risk disclosure as they provide the institution, investors, and others with the information necessary to understand the risks faced by that institution and, over time, how successfully the institution is addressing those risks. While target-setting has become increasingly important for financial institutions committed to realigning their business with a net-zero emissions pathway, there is currently no such pathway for target-setting against mitigating physical climate risks or alignment with adaptation goals.

**[3]** TNFD (Task Force on Nature-related Financial Disclosures) recommends that companies disclose on the full set of nature-related dependencies, impacts, risks and opportunities (including climate) of their operations and across their value chain. This includes a consideration of the upstream (supply) and downstream (distribution and sale) value chains. For financial institutions, this includes lending, investment and/or insurance, as well as fee-based advisory activities. The TNFD also suggests assessing nature-related risks and opportunities over medium- to long-term time frames and requires a consideration of a broader set of dependencies and impacts, as these may lead to additional risks and opportunities that are material for enterprise value over time.

TNFD understands that nature-related risk management and disclosure will be new to many companies. Thus, firms may wish to start by prioritizing their disclosures and focus on specific activities or business lines where such information is material. This might, for example, include focusing on specific geographic areas, aspects of their value chain as well as specific impact drivers, nature impacts and nature-related dependencies. For financial firms they may wish to focus on certain asset classes or portions of their financing and advisory activities. In the TNFD beta framework, it is specified that users should be clear what was considered in scope for their disclosure and what has not been considered for the scope of their disclosure. Recognizing that this will be a journey for most organizations as their awareness of, and capabilities for managing nature-related risks increases, disclosure coverage should expand over time so that after no more than five years firms are considering their full set of material dependencies, impacts, risks and opportunities across their upstream and downstream operations.

Aligned with the TCFD approach, TNFD believe scenario analysis can play an important role in informing the strategy, governance, risk management and capital allocation decisions of companies and financial institutions. Recognizing the complex interplay of nature-related dependencies and impacts an organization has over the short, medium, and long term, the TNFD's draft disclosure recommendations specify that risks should be assessed taking into consideration different scenarios (plausible futures) and the implications for nature-related physical, transition and systemic risks and opportunities.

The complexity of data required for ESG, TCFD, TNFD and pro-innovation sustainable investments' is structured and presented in Figure 1.

### 2.3 Comparing synthetic data options for generic and sustainable finance

Synthetic data in finance refers to artificially generated data that is not sourced from real-world financial events but shares the same statistical properties. There is a number of diverse applications for synthetic data in finance, and while some applications of synthetic data in sustainable finance overlap with generic finance, there are nuances and specificities related to ESG factors (Table 1) (Zhang and Chen, 2017; Papacharalampopoulos et al., 2020; Ljung, 2021; Popescu et al., 2021; Horvath, 2022; Valle-Cruz et al., 2022; Kelly et al., 2023; Sauer et al., 2023).

**Table 1:** Identified cases of synthetic data in generic finance, their interpretation within sustainable finance and presentation of select methodologies for corresponding use domains.

**Applications in finance**	**Relevance for sustainable finance**	**Methods in the literature**
Asset management	For investments in agriculture, forestry, or real estate, synthetic data can model future land use scenarios, crop yields, or urban development trajectories, aiding in investment decision-making. For green infrastructure projects like wind farms or solar parks, synthetic remote sensing data can help visualize future scenarios, like the impact of vegetation growth on a solar park's efficiency.	Time Series Analysis (ARIMA, GARCH, and cointegration), Machine Learning Models (Random Forests, Support Vector Machines, and Gradient Boosting Machines), Optimization Algorithms (Markowitz model or Black-Litterman model), Reinforcement Learning (Q-Learning and Deep Reinforcement Learning), VaR Models (Monte Carlo simulations, Historical Simulation, or parametric methods), Natural Language Processing [Sentiment scores, Topic modeling (LDA)], High-Frequency Trading Algorithms, Deep Learning [recurrent neural networks (RNNs) and long short-term memory networks (LSTMs)], Clustering Algorithms (K-means or hierarchical clustering), Isolation Forests (specialized tree-based method designed for anomaly detection in higher dimensions), Principal Component Analysis, SHAP (SHapley Additive exPlanations).
Algorithm testing	Algorithms may be developed to evaluate and predict ESG performance or to automatically sort investments based on ESG criteria. Synthetic data helps in testing these algorithms in diverse scenarios. In the absence of historical remote sensing data, synthetic data can serve as a benchmark, helping validate models or algorithms designed to interpret recent remote sensing data.	Generative Adversarial Networks (GANs), Monte Carlo Simulations.
Risk management	Use synthetic data to model potential future risks associated with climate change, political shifts toward sustainability, or social unrest. By simulating potential environmental disasters like flooding, droughts, or wildfires using synthetic data, financial institutions can assess the risks associated with investments in vulnerable regions.	Monte Carlo Simulations, Copula-based methods (generate multivariate synthetic datasets preserving the dependencies among variables).
Data privacy and security	Sustainable finance may require specific ESG-related data sets that are less commonly available, making their protection crucial. In regions where there are restrictions on capturing or sharing real remote sensing data due to security concerns, synthetic data can provide a viable alternative for analysis without compromising security.	Differential Privacy (adds noise to data in a way that protects individual data points), Data Masking (replaces sensitive information with modified content (characters or values) but structurally similar to the original data).
Data augmentation	ESG data is often sparse, especially from companies in emerging markets or newer industries, and synthetic data can fill these gaps. When real remote sensing datasets are limited, synthetic datasets can augment the training data, improving the performance of machine learning models used for analyzing and interpreting satellite images.	Bootstrap Resampling, SMOTE (Synthetic Minority Over-sampling Technique)
Market monitoring	Synthetic remote sensing data can simulate potential environmental changes, helping investors understand how specific areas might be affected by climate change, deforestation, or other environmental factors.	ARIMA, GARCH, Random Forests, SVMs, Neural Networks & Reinforcement Learning, NLP (sentiment analysis, topic modeling, event extraction), Clustering Algorithms (K-means, DBSCAN), Isolation Forests & Decision Trees, Regression Analysis, VaR (Value at Risk), TF-IDF (Term Frequency-Inverse Document Frequency, for information retrieval in documents like SEC filings), Graph Algorithms, Bayesian Networks, Bootstrap Aggregating (Bagging) & Boosting, Mean-Variance Optimization & Black-Litterman Model.
Regulatory compliance	Regulations might involve meeting specific ESG targets or reporting standards. Synthetic data can help test compliance under hypothetical scenarios. Companies and investors can use synthetic data to visualize and communicate potential future environmental impacts or benefits of their investments, enhancing transparency and stakeholder trust.	Agent-based modeling, Scenario generators.
Credit scoring	Credit models may incorporate ESG factors, predicting a company's future performance based on its sustainability initiatives. Synthetic data can help with training these models.	GANs, Decision Trees and Random Forests.
Cost efficiency	Generating synthetic remote sensing data can be more cost-effective than launching new satellite missions or frequently flying drones, especially when testing hypotheses or models.	Regression Analysis, Classification Algorithms, Random Forests, Support Vector Machines, Neural Networks, ARIMA (AutoRegressive Integrated Moving Average), GARCH (Generalized Autoregressive Conditional Heteroskedasticity), NLP (Sentiment Analysis & Topic Modeling), Q-learning and Deep Q Networks, Policy Gradients, K-means, Hierarchical Clustering, GANs (Generative Adversarial Networks), Autoencoders, Linear Programming, Genetic Algorithms, Monte Carlo Simulations, Bagging/Boosting/Stacking, Decision Trees like CART (Classification and Regression Trees), Bayesian Networks.
AML/Fraud detection	Focus on detecting ‘greenwashing' (where companies falsely claim sustainable practices). Synthetic data can simulate such activities for better detection.	SMOTE [generates synthetic examples of underrepresented classes (like fraud instances)], Bayesian Networks.
Product development	Develop financial products targeting green investments or ESG compliant portfolios. Synthetic data can simulate market responses to such products.	Agent-based modeling, Variational Autoencoders (can generate new customer profiles and behaviors).
Financial education and training	Training focuses on understanding ESG risks and opportunities. Synthetic data can simulate potential future ESG scenarios.	Time-series simulations (generate synthetic data streams resembling market data), Rule-based systems (create scenarios based on predefined rules and principles).
Scenario analysis	Emphasis on predicting future scenarios related to climate change, societal shifts, and governance changes. Synthetic remote sensing data can help simulate how different ESG factors might impact landscapes, such as how sustainable agricultural practices influence soil health and vegetation over time.	Monte Carlo Simulations & Stochastic models.

Addressing these data gaps is crucial for investors, regulators, and other stakeholders to make informed decisions in the realm of sustainable finance (Santos et al., 2021; Behera et al., 2022; Rojas-Hernández, 2023). As the sector evolves, there's a growing push for standardizing ESG reporting and improving data transparency. Generating synthetic data for the missing data types in sustainable finance requires specialized techniques tailored to the nature of each data category (Moro-Visconti et al., 2020; Chatterjee and Byun, 2023; Pawlik and Dziekański, 2023).

Thus, in generic finance traders and investment managers start utilizing synthetic data to test new trading algorithms, ensuring they are robust across a variety of market conditions, including those that have not been yet experienced, and in cases where real data is sparse, synthetic data can supplement the dataset to improve the performance and training of machine learning models. Financial institutions can create synthetic versions of sensitive data sets, allowing external researchers or developers to work on projects without risking the exposure of confidential information, whilst regulators can test the impact of new policies or regulations using synthetic data to avoid unintended consequences in the real market. From the risk management perspective, synthetic data can be helpful in simulating extreme market conditions, enabling institutions to assess their resilience to shocks and stress-test their portfolios. Finally, by generating synthetic profiles of borrowers, financial institutions can also improve the models they use to assess credit risk, especially for underrepresented or new-to-credit populations. Missing data in sustainable finance can pose challenges (Bonnéry et al., 2019; Campbell, 2019; Hosaka, 2019; Beery et al., 2020, 2021; Koh et al., 2020; Kuchin et al., 2020; Beery, 2021; Dietz et al., 2021; Kahn et al., 2021; Norouzzadeh et al., 2021; Van Horn et al., 2021; Warren et al., 2021; Ziolo et al., 2021; Azamuke et al., 2022; Barnes, 2022; Walsh et al., 2022; Kannan and Nandwana, 2023), especially given the sector's emphasis on comprehensive analysis and decision-making based on Environmental, Social, and Governance (ESG) criteria. However, ESG and associated regulatory data requirements within sustainable finance sub-domain are also seen to extend toward more ‘pro-innovation' use-cases, as green and transition investments are developing and maturing from incubator phases toward mainstream products. The types of missing data in sustainable finance can be therefore matched against the relevant mainstream finance categories (examples are presented in Table 1).

## 3 Ethical considerations for synthetic data deployments

The advent of generative artificial intelligence (AI) technologies has ushered in groundbreaking capabilities for synthetic data generation. While these capabilities offer promising advantages in various sectors, they simultaneously give rise to complex ethical and societal dilemmas. A particularly concerning attribute of generative AI is its ‘self-replicating' nature, which often relies on unstructured, multi-modal datasets to generate further synthetic data. As these datasets rapidly deplete, the ethical quandaries surrounding synthetic data come to the fore (Alemohammad et al., 2023).

In this context two major applications of synthetic data include its role as an output of generative AI models and as an input. In the first scenario, synthetic data serves crucial functions in sectors like banking where privacy and ethical considerations hinder data availability for tasks such as training environments and scenario testing. In the second instance, synthetic data compensates for minority classes in datasets needed for critical applications, such as fraudulent transactions or ‘greenwashing', thereby usefully augmenting training data for machine learning models.

However, the ethical challenge still remain in validating the utility, fidelity, and privacy of synthetic data. Validation remained a significant barrier to its broader adoption (Battese et al., 1988; Fienberg, 1994; Drechsler, 2011; Warmenhoven et al., 2020; James et al., 2021; Keen, 2021; Peachey et al., 2021; White, 2021; Heim, 2022; Krenchel and Cury, 2022). Assessing specific requirements of a use case is pivotal in evaluating both the utility and privacy concerns surrounding synthetic data. While model generalizability might increase the utility of synthetic data across multiple use cases, it poses ethical risks related to model drift and re-identification of individuals in the dataset.

For a more ethical deployment of synthetic data, mathematical validation methods for the generative model need to be augmented by post-generation validation techniques. Industry adoption may benefit from a shift toward a risk-based model for privacy validation that acknowledges some level of inherent risk in the synthetic data generation and sharing process. Moreover, the ethical adoption of synthetic data can be facilitated through comprehensive use-case documentation, development of standardized frameworks, and regulatory guidance.

Thus, while synthetic data presents an invaluable resource for modern AI applications, a multidisciplinary approach involving ethical considerations, mathematical validation, and industry standardization is essential for its responsible adoption.

## 4 Conclusions and discussion

After the thorough analysis of research literature, it can be concluded that synthetic data has the potential to revolutionize ethical applications in finance by providing a means to conduct robust analyses without compromising values of the highly (or not highly) confidential datasets. By generating artificial data that mimic real financial patterns, synthetic data enables institutions to sidestep the ethical pitfalls associated with using sensitive customer, business or other corporate value information, thereby ensuring compliance with current regulations and thus enhancing the integrity of financial models.

In the burgeoning field of sustainable finance, synthetic data opens up a wealth of opportunities, addressing the sector's voracious appetite for data to support compliance, screening, and proactive investment decisions. With an increasing emphasis on ESG (Environmental, Social, Governance) criteria, financial institutions require extensive datasets to evaluate the sustainability of investments and to monitor the social and environmental impact of their portfolios. Synthetic data can provide high-quality, scalable information, facilitating the development of innovative financial products and strategies. As a tool, it enhances risk assessment models by incorporating potential ESG scenarios, allowing for stress testing against a range of sustainability factors.

However, deploying synthetic data in sustainable finance is not without challenges. Concerns over the representativeness of synthetic datasets can lead to questions about the reliability of insights derived from them. Moreover, the complexity of ESG variables demands synthetic data that is sophisticated enough to accurately reflect the nuanced interplay of these factors. Overcoming these obstacles requires advances in algorithmic techniques to ensure that synthetic data retains the intricate correlations present in genuine data. It also necessitates rigorous validation processes to establish the credibility of the synthesized datasets, thus paving the way for their effective application in driving sustainable finance forward.

## Data availability statement

The raw data supporting the conclusions of this article will be made available by the authors, without undue reservation.

## Author contributions

NT conceived the idea, completed literature reviews, and wrote up the manuscript.

## Footnotes

^1^ https://www.sec.gov/

^2^ https://www.cftc.gov/

^3^ https://www.canada.ca/en/financial-consumer-agency.html

^4^ https://asic.gov.au/

^5^ https://www.esma.europa.eu/

^6^ https://www.mas.gov.sg/

^7^ https://www.sebi.gov.in/

^8^ http://www.csrc.gov.cn/csrc_en/index.shtml

^9^ https://www.fsa.go.jp/en/

^10^ https://www.fsca.co.za/Pages/Default.aspx

^11^ https://www.gov.br/cvm/en

^12^ https://www.fca.org.uk/firms/innovation/engagement/iag

^13^ https://www.fca.org.uk/news/news-stories/launch-permanent-digital-sandbox

^14^Concurrent with the release of its 2023 status report on October 12, 2023, the TCFD has fulfilled its remit and disbanded. The FSB has asked the IFRS Foundation to take over the monitoring of the progress of companies' climate-related disclosures.
